# Targeting mitotic chromosomes: a conserved mechanism to ensure viral genome persistence

**DOI:** 10.1098/rspb.2008.1642

**Published:** 2009-01-20

**Authors:** Katherine M. Feeney, Joanna L. Parish

**Affiliations:** Bute Medical School, University of St AndrewsBute Building, St Andrews, Fife KY16 9TS, UK

**Keywords:** viral persistence, segregation, DNA tumour virus, genome, mitosis

## Abstract

Viruses that maintain their genomes as extrachromosomal circular DNA molecules and establish infection in actively dividing cells must ensure retention of their genomes within the nuclear envelope in order to prevent genome loss. The loss of nuclear membrane integrity during mitosis dictates that paired host cell chromosomes are captured and organized by the mitotic spindle apparatus before segregation to daughter cells. This prevents inaccurate chromosomal segregation and loss of genetic material. A similar mechanism may also exist for the nuclear retention of extrachromosomal viral genomes or episomes during mitosis, particularly for genomes maintained at a low copy number in latent infections. It has been heavily debated whether such a mechanism exists and to what extent this mechanism is conserved among diverse viruses. Research over the last two decades has provided a wealth of information regarding the mechanisms by which specific tumour viruses evade mitotic and DNA damage checkpoints. Here, we discuss the similarities and differences in how specific viruses tether episomal genomes to host cell chromosomes during mitosis to ensure long-term persistence.

## 1. Introduction

**Figure 1:**
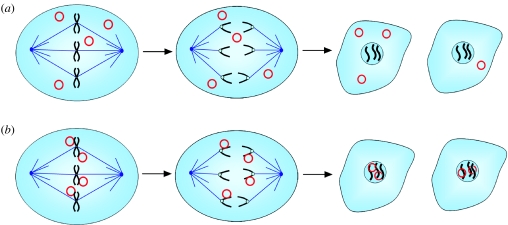
Genome tethering during mitosis ensures nuclear retention and viral persistence. (*a*) In the absence of a tethering mechanism, episomally maintained DNA molecules (red circles) are potentially partitioned unevenly and excluded from the nuclear compartment upon completion of mitosis. This leads to a non-productive and transient viral infection. (*b*) A robust tethering mechanism ensures that episomal DNA molecules are more evenly distributed between daughter cells and remain in the nuclear compartment, thus ensuring genome maintenance in dividing cells and persistent viral infection.

**Figure 2:**
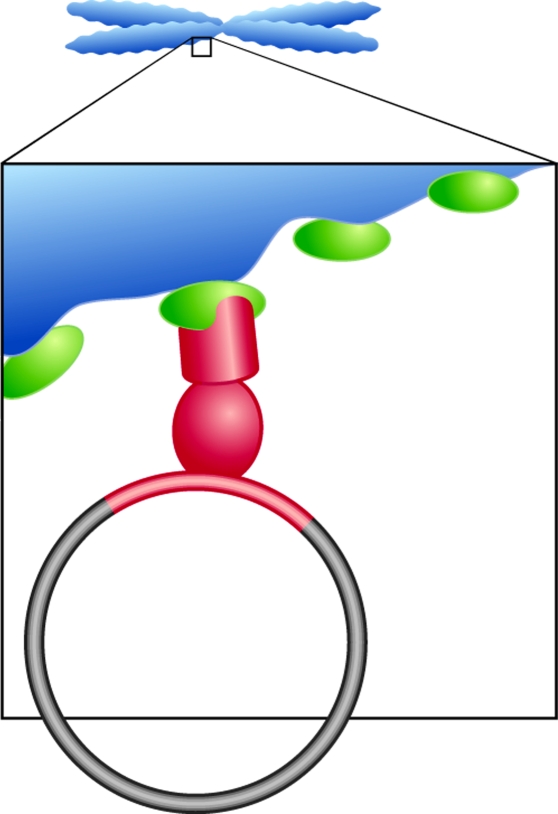
Mechanism of viral genome tethering during mitosis. To ensure persistence in dividing cells, many episomally maintained DNA viruses tether genomes to host cell chromosomes during mitosis. In general, a virally encoded DNA-binding protein (red) associates with specific sequences within the viral genome (circle) while simultaneously associating with chromatin (blue)-bound cellular protein(s) (green). This intricate tethering mechanism appears conserved among diverse viral types.

While the specific mechanism of genome segregation during mitotic cellular division employed by each DNA virus studied has subtle or sometimes major distinctions, the overall theme of the way in which viruses have evolved to ensure genome persistence in dividing cells remains strikingly similar. Viral genomes are replicated along with the cellular DNA and are attached to host cell chromosomes during cellular division. This ensures retention in the nuclear envelope and in some cases may facilitate a roughly even distribution of episomal DNA molecules to each daughter cell (figure 1). To facilitate this mechanism of genome tethering, each virus encodes a DNA-binding protein, which specifically associates with repeated sequences within the viral genome. The viral DNA-binding protein then targets host cell proteins that themselves associate with mitotic chromatin, thus tethering viral genomes to the host cell DNA during mitotic segregation (figure 2). This elegant mechanism appears to have evolved to ensure viral persistence during low copy latent infections, and thus provides a powerful and novel antiviral target. The similarities and differences in the way in which different DNA tumour viruses have developed their own segregation mechanism will be discussed in this review.

## 2. Epstein–Barr virus

Epstein–Barr virus (EBV) is a human γ-herpesvirus (genus lymphocryptovirus) that persists benignly in approximately 90 per cent of individuals worldwide. In developing countries, infection occurs in early childhood and is generally asymptomatic. Owing to better hygiene standards, infection is delayed until early adolescence in developed countries where it can result in infectious mononucleosis (Rickinson *et al.* 2000). Persistent EBV infection is tightly linked to the development of several human cancers including Burkitt's lymphoma, Hodgkin's disease and nasopharyngeal carcinoma (Crawford 2001). Although EBV potently transforms cells in culture, it is known to persist *in vivo* not by immortalizing infected B cells, but by establishing a true latency in normal resting memory B cells (Thorley-Lawson 2005).

During a latent infection, the EBV genome is episomally maintained as a 165 kb double-stranded circular DNA molecule, where nine latent proteins including five EBV nuclear antigens (EBNA) and three latent membrane proteins are expressed. These proteins are required to stimulate proliferation of the host cell and maintain a stable viral copy number within dividing cells (Thorley-Lawson 2005). Replication of the viral genome is stimulated once per cell cycle (Adams 1987) and requires repeat sequences within the viral origin of replication *OriP* and the viral EBNA1 protein (Lupton & Levine 1985; Reisman *et al.* 1985; Yates *et al.* 1985, 2000; Middleton & Sugden 1992). *OriP* contains 20 high-affinity 18 bp EBNA1-binding sites within the family of repeats (FR) element and four low-affinity binding sites within the dyad symmetry (DS) element (Reisman *et al.* 1985). Through its specific affinity for sequences within *OriP*, EBNA1 is also essential for transcriptional control of viral enhancers and promoters (Reisman & Sugden 1986; Sugden & Warren 1989; Wysokenski & Yates 1989; Altmann *et al.* 2006), and the retention of the viral genomes in dividing cells (Middleton & Sugden 1994; Langle-Rouault *et al.* 1998).

The viral genome partitioning function of EBNA1 has been directly attributed to its ability to associate with host cell chromosomes during mitosis. EBNA1 localizes to discrete foci during mitosis that colocalize with host cell mitotic chromosomes (Grogan *et al.* 1983; Petti *et al.* 1990). Deletion studies have demonstrated that three independent domains of EBNA1 are involved in mitotic chromosome binding. Each of these domains are rich in basic residues and it is suggested that these regions may assist in chromosomal attachment via interaction with histone H1 (Marechal *et al.* 1999). Further work has shown that EBNA1 targets the cellular protein EBNA1 binding protein 2 (EBP2) to facilitate genome partitioning (Wu *et al.* 2000; Kapoor *et al.* 2001; Kapoor & Frappier 2003). The interaction between EBNA1 and EBP2 was originally identified in a yeast two-hybrid screen (Shire *et al.* 1999), and the requirement of EBP2 for the association of EBNA1 with chromosomes and EBNA1-dependent segregation of FR-containing plasmids was demonstrated using a reconstituted yeast system (Kapoor *et al.* 2001; Kapoor & Frappier 2003). In addition, EBNA1 and EBP2 have been shown to colocalize on mitotic chromosomes in human cells and deletion of one of the chromosome-binding sites identified by Marechal *et al.* (1999) results in a loss of association of EBNA1 with EBP2 (Wu *et al.* 2000). Interestingly, it has been demonstrated that the association of human EBP2 with chromosomes is regulated by the Aurora family kinase member Aurora B (Kapoor *et al.* 2005). This suggests a role for the mitotic checkpoint regulator Aurora B in the partitioning of EBV genomes, although EBP2 itself is not thought to be a direct target of Aurora kinase.

In contrast to the studies described above, it has been suggested that EBNA1 associates directly with mitotic chromosomes in human cells independently of its association with EBP2 (Lindner & Sugden 2007). This interaction is proposed to be via an AT-hook motif within EBNA1 that binds to adenosine/thymidine (A/T)-rich DNA sequences (Sears *et al.* 2004). Similar to the EBP2-binding region within EBNA1, the two EBNA1 AT-hook motifs overlap with chromosomal binding regions described by Marechal *et al.* (1999), and it has been shown that the partitioning function of EBNA1 correlates with its AT-hook activity and not with EBP2 binding (Sears *et al.* 2004). In support of this, fusion of the DNA binding and dimerization domain of EBNA1 that lacks the EBP2-binding region, with cellular proteins containing AT-hook motifs, supports wild-type levels of viral plasmid replication and partitioning (Altmann *et al.* 2006).

Mutant EBNA1 proteins that fail to activate transient replication of *OriP*-containing plasmids are also defective in genome tethering function (Kanda *et al.* 2001). Therefore, it has been suggested that EBNA1 functions to couple viral genome replication during S-phase to the subsequent tethering of replicated genomes to chromosomes during mitosis. In support of this, it has been demonstrated that replicated *OriP*-containing plasmids are spatially colocalized as pairs on mitotic chromosomes and that this positioning is essential for their non-random partitioning (Kanda *et al.* 2007; Nanbo *et al.* 2007). Nanbo *et al.* (2007) speculated that EBNA1 associates with chromosomal DNA during G1 and that replication of a subset of viral genomes is coincident with progression to the S-phase. This results in an association of the newly replicated EBV sister plasmids with the same or nearby sites on each sister chromatid where the cellular cohesin complex (Nasmyth 2005) may be required to hold the tethered EBV plasmids together. This model proposes that the replicated EBV sister plasmids are segregated to daughter cells as the sister chromatids separate at anaphase and offers an exciting twist to the specific mechanism EBV uses to maintain low copy number episomal genomes in latent and persistent viral infections.

## 3. Rhadinovirus

### (a) Kaposi's sarcoma-associated herpesvirus

Similar to EBV, Kaposi's sarcoma-associated herpesvirus (KSHV; also known as human herpesvirus 8, HHV-8) is a γ-herpesvirus. KSHV belongs to the genus Rhadinovirus and is the only member of the genus known to infect humans. The incidence of KSHV infection is low in Asia, northern Europe, Australia and the Americas (less than 5%) but higher in Mediterranean countries (4–35%) and sub-Saharan Africa (20–70%) (Schulz *et al.* 2002). KSHV infection is typically asymptomatic in immunocompetent individuals. Nonetheless, KSHV is the causative agent of primary effusion myeloma, Castleman's disease and Kaposi's sarcoma (Moore & Chang 2001). Kaposi's sarcoma, which is characterized by pigmented sarcomas of the skin, is often the presenting symptom of acquired immune deficiency syndrome (AIDS). In fact, KSHV was discovered by the analysis of DNA fragments from AIDS-associated Kaposi's sarcoma (Chang *et al.* 1994).

The KSHV genome comprises a long unique region (140 kb) flanked by two terminal repeat (TR) regions, which consist of tandem repeats of 803 bps long (Russo *et al.* 1996). The number of repeated sequences varies between strains (Judde *et al.* 2000). During latent infection, only a relatively short portion (11 kb) of the viral episome is transcribed. These latent transcripts encode six proteins; viral cyclin D, latency-associated nuclear antigen 1 (LANA1/LANA), Kaposin A and B, viral FAS ligand interleukin-1B-converting enzyme inhibitory protein, and the viral homologue of interferon regulatory factor 3 (LANA2); and 12 pre-miRNAs (reviewed in Laurent *et al.* 2008). To date, 86 open reading frames (ORFs) have been identified and possibly more exist as further splice variants are discovered.

LANA protein is present in nearly all infected cells, and therefore, anti-LANA antibodies can be used as a marker of infection (Rainbow *et al.* 1997; Laney *et al.* 2006). LANA, encoded by ORF73, plays multiple roles in the viral life cycle and thus is the most extensively studied latent transcript. LANA is required for viral replication, episome maintenance and regulation of latent gene expression. It has been clearly demonstrated that LANA promotes progression through the cell cycle by interacting with p53, pRb and c-Myc pathways (Friborg *et al.* 1999; Radkov *et al.* 2000; Liu *et al.* 2007). Similar to EBV EBNA1 and the papillomavirus E2 protein (discussed below), disruption or depletion of LANA results in reduced episomal maintenance (Ye *et al.* 2004; Godfrey *et al.* 2005).

LANA facilitates viral DNA replication via a specific interaction of the C-terminal DNA-binding domain with two sites within the TR: LANA-binding site 1 (LBS1) and LANA-binding site 2 (LBS2) (Garber *et al.* 2002; Hu *et al.* 2002). Computational analysis shows that the C-terminus is similar in structure to the EBV EBNA1 DNA-binding domain (Grundhoff & Ganem 2003). In addition, the LBS1/2 regions are similar in organization to the EBV DS element and binding of LANA and EBNA1, respectively, is required for DNA replication (Rawlins *et al.* 1985; Yates *et al.* 1985; Polvino-Bodnar & Schaffer 1992; Garber *et al.* 2002; Hu *et al.* 2002; Lim *et al.* 2002; Hu & Renne 2005). It has recently been shown that LANA binds to host cell origin recognition complexes (ORCs) and may recruit ORCs to sites of latent origins of replication (Stedman *et al.* 2004; Verma *et al.* 2006).

Almost a decade ago, two independent research groups suggested a role for LANA in the tethering of viral episomes to host chromosomes during mitosis (Ballestas *et al.* 1999; Cotter & Robertson 1999). As previously mentioned, LANA binds to the TR via its C-terminus (Ballestas & Kaye 2001; Cotter *et al.* 2001; Komatsu *et al.* 2004). The residues within the C-terminus, which are required for TR DNA binding, have been mapped in some detail, and correspond to those required for EBNA1 DNA binding (Kelley-Clarke *et al.* 2007). The mechanism by which LANA associates with chromosomes remains to be elucidated. LANA has a punctate nuclear distribution in the presence of the KSHV genome or TR-containing plasmids but in the absence of TR DNA, LANA is distributed diffusely in the nucleus (Ballestas *et al.* 1999; Schwam *et al.* 2000). Although both the N- and C-termini can bind to chromosomes, it seems that LANA is recruited to TR DNA via binding of the C-terminal domain while the N-terminus tethers episomes to chromosomes by binding chromosome-associated proteins (Piolot *et al.* 2001; Barbera *et al.* 2006).

Several cellular proteins have been implicated in the genome tethering mechanism of KSHV. Recruitment of LANA to mouse chromosomes has been shown to require methyl CpG-binding protein (MeCP2) and DEK (Krithivas *et al.* 2002). However, a more recent study illustrates that the LANA residues responsible for chromosome binding interact with histones H2A and H2B (Barbera *et al.* 2006). Perhaps MeCP2 and DEK, with their associated chromatin-modifying properties, facilitate LANA binding to nucleosomes (Bowen *et al.* 2004; Waldmann *et al.* 2004). In addition to the aforementioned proteins, LANA has also been shown to interact with Brd4, Brd2/Ring3, Histone H1 and nuclear mitotic apparatus protein (NuMA; Cotter & Robertson 1999; Ottinger *et al.* 2006; You *et al.* 2006; Si *et al.* 2008). In addition to the cellular proteins directly targeted by LANA, it has been shown that the cellular cohesin complex and the transcriptional insulator CCCTC-binding factor (CTCF) associate with sequences within the latency control region of KSHV genomes, and that together these factors play a role in the control of latent gene expression (Stedman *et al.* 2008). The authors also speculate that, similar to the related EBV, KSHV genomes may remain associated following replication and that genomes are actively segregated like host cell chromosomes by the regulation of cohesin association. Interestingly, CTCF has also been shown to associate with EBV episomes, although this is thought to be required for the transcriptional regulation of viral genes by facilitating chromatin domain organization, and not for genome maintenance *per se* (Chau *et al.* 2006; Day *et al.* 2007). It is clear that further research is required to dissect the contribution of individual cellular proteins to the maintenance of episomal KSHV genomes.

### (b) Other ORF73-encoded proteins

Almost all γ_2_-herpesviruses encode homologous proteins from ORF73. The proteins vary greatly in length; KSHV LANA is one of the longest (1162 amino acids) while bovine herpesvirus 4 (BoHV-4) ORF73 is the shortest (253 amino acids). The acidic internal repeat region is the least conserved region and varies in length and in some species is completely absent (murine γ-herpesvirus 68 (MHV-68), rhesus rhadinovirus (RRV) and BoHV-4). Conversely, the N- and C-terminal regions of proteins translated from ORF73 are highly conserved (Burnside *et al.* 2006; Taus *et al.* 2007). Similar to LANA, herpesvirus saimiri (HVS) ORF73 inhibits p53 and pRb function and it has been shown that the expression of MHV-68 ORF73 results in decreased p53 stabilization (Borah *et al.* 2004; Forrest *et al.* 2007).

There is some evidence to suggest that the genome tethering function of proteins expressed from ORF73 is also conserved. HVS ORF73 has been shown to tether the viral genome to host chromosomes and facilitate episomal maintenance by binding to TR DNA (Collins *et al.* 2002; Verma & Robertson 2003; White *et al.* 2003). Furthermore, the C-terminus of HVS ORF73 is required for nuclear localization, a feature which is also characteristic of KSHV LANA (Hall *et al.* 2000). There have been a limited number of studies on MHV-68 ORF73; however, it has been demonstrated that ORF73 is required to establish and maintain latency (Fowler *et al.* 2003; Moorman *et al.* 2003). It has also been shown that LANA can regulate viral latency by inhibiting the replication and transcription activator protein (RTA), which is encoded by ORF50 and functions as a transcriptional activator of early and late lytic genes (Lan *et al.* 2004). Similarly, HSV ORF73 blocks RTA-mediated transactivation by direct inhibition of ORF50 expression (Schafer *et al.* 2003). RRV ORF73 also represses RTA-mediated transactivation, although the mechanism is unclear (DeWire & Damania 2005). This provides evidence that both HVS- and RRV ORF73-encoded proteins inhibit ORF50 function. Further evidence for conservation of the lytic switch mechanism is demonstrated by the fact that KSHV RTA can reactivate MHV-68 from latency (Rickabaugh *et al.* 2005).

## 4. Papillomavirus

Papillomaviruses are a diverse group of small DNA viruses that infect epithelial cells with numerous clinical outcomes. There are approximately 120 viral types described that infect mammals and birds with a high degree of species specificity. Of these, approximately 100 human papillomavirus (HPV) types have been defined, which infect the basal cells of either mucosal or cutaneous epithelia in a variety of biological niches (de Villiers *et al.* 2004). Once infection is established, papillomaviruses can persist in infected individuals for long periods of time and many types appear to have a latent life cycle where viral DNA can be detected in areas of healthy skin (Antonsson & Hansson 2002; Antonsson *et al.* 2003). HPV infection commonly causes benign tumours (warts and papillomas) but is often associated with malignant progression including cervical, anogenital or oropharyngeal carcinomas. HPVs are subdivided into five genera depending on the preferential site of infection and clinical outcome. The two largest HPV genera are the alpha and beta papillomaviruses and comprise 90 per cent of all known HPV types. The association of specific HPV types with carcinoma is used to further classify viral types. For example, within the alpha or genital/mucosal papillomavirus group, HPV types 6 and 11 are classified as low-risk and have little or no association with anogenital carcinoma whereas infection at the same site with high-risk HPVs, such as types 16 and 18, can result in malignant progression. In support of this, it has been shown that more than 99.7 per cent of cervical cancers contain high-risk HPV DNA with approximately 50 per cent of these containing HPV-16 DNA (Doorbar 2006).

All papillomaviruses contain an approximately 8000 bp double-stranded circular DNA genome that encodes eight or nine ORFs including six early ORFs (E1, E2, E4, E5, E6 and E7) and two late ORFs (L1 and L2). Upon infection of basal cells within the epidermis, the viral genome is established as a stable episome and maintained at a low copy number. Viral genomes are replicated along with the cellular DNA during the S-phase, which requires the viral E1 and E2 proteins. E2 is a multifunctional DNA-binding protein that specifically recognizes the consensus motif (AACCG(N4)CGGTT) (Dell *et al.* 2003), which is repeated multiple times in the non-coding regulatory region or long control region (LCR) of the viral genome. The association of E2 with binding motifs within this region is necessary for the recruitment of the E1 helicase, which in turn melts the origin of replication and recruits cellular proteins required for replication, such as replication protein A and DNA polymerase α primase (Masterson *et al.* 1998; Conger *et al.* 1999; Han *et al.* 1999; Loo & Melendy 2004). E2 also functions as a transcription factor and regulates the expression of the E6 and E7 viral oncogenes by association with motifs within the LCR that lie close to the early promoter.

In addition to its role in viral genome replication and transcriptional regulation, E2 is required for the stable maintenance of viral genomes by facilitating an intricate segregation mechanism of viral genomes in dividing cells. This mechanism has been extensively studied in both human and animal papillomaviruses with subtle differences between genera reported. Initial studies were carried out using bovine papillomavirus type 1 (BPV-1). The BPV-1 E2 protein associates with mitotic chromosomes throughout mitosis in a manner that requires sequences within the N-terminal transactivation domain of E2 (Skiadopoulos & McBride 1998; Ilves *et al.* 1999; Bastien & McBride 2000; Abroi *et al.* 2004). From this, it was hypothesized that the E2 protein from all papillomavirus types form a link between viral genomes, specifically bound by the C-terminal DNA-binding domain of E2, and mitotic chromosomes, bound by the N-terminal transactivation domain of E2 via interaction with cellular chromatin-associated protein(s). This is consistent with the segregation mechanism of the other DNA viruses described in this review, and has remained the common theme for all HPVs tested with some subtle differences.

While BPV-1 E2 stably associates with chromosomes throughout mitosis in numerous random foci, the E2 proteins from the alpha group (e.g. HPV-11, -16, -31 and -57) have been shown to stably interact with mitotic chromosomes only in early (prophase) and late (telophase) mitosis, but not at the point of chromosomal separation (anaphase) (Oliveira *et al.* 2006; Donaldson *et al.* 2007). However, it was noted that the interaction of these E2 proteins with chromosomes throughout mitosis can be observed if cells are pre-extracted prior to fixation (Oliveira *et al.* 2006), suggesting that the interaction of alpha group E2 proteins with mitotic chromosomes may occur but is highly dynamic and unstable. The HPV-8 E2 protein displays further differences in its mitotic localization pattern in that it appears to associate with chromosomes only at sites very close to the point of attachment of the mitotic spindle. This is potentially in agreement with a study by Van Tine *et al*. (2004) which showed that plasmids containing E2-binding sites are localized in foci adjacent to the spindle attachment site in an HPV-11 E2-dependent manner, although further studies from the same group have suggested that the HPV-11 E2 protein itself associates with the spindles during mitosis (Van Tine *et al.* 2004; Dao *et al.* 2006). However, the biological significance of this remains unclear.

The search for the specific mechanism by which E2 tethers viral genomes to mitotic chromosomes has been long standing, and the cellular proteins that E2 targets to facilitate segregation of genomes remain unclear. It has been reported that the bromodomain family member Brd4 is required for the attachment of HPV-31, HPV-16 and BPV-1 E2 to mitotic DNA (You *et al.* 2004; Baxter *et al.* 2005; Abbate *et al.* 2006). In support of this, Brd4 and BPV-1 E2 colocalize at punctuate foci on mitotic chromosomes and overexpression of the C-terminal domain (Brd4–CTD) or a small peptide shown to block the interaction between E2 and Brd4 prevents the association of E2 with host cell chromosomes and enhances genome loss in BPV-1-transformed cells (McPhillips *et al.* 2005; You *et al.* 2005; Abbate *et al.* 2006). While these data suggest a role for Brd4 in the tethering of E2 to host cell chromosomes, it has also been demonstrated that Brd4 associates with E2 to facilitate E2-dependent transcriptional regulation (Ilves *et al.* 2006; McPhillips *et al.* 2006; Schweiger *et al.* 2006; Senechal *et al.* 2007; Wu & Chiang 2007), and that Brd4 is not required for the maintenance of all papillomavirus types (McPhillips *et al.* 2006). In support of this, mutant E2 proteins that fail to associate with Brd4 and do not support stable genome maintenance are unable to activate transcription (Baxter *et al.* 2005; Abbate *et al.* 2006; Ilves *et al.* 2006). This suggests that inhibition of Brd4 association is unlikely to only affect chromosome attachment, but will also alter other important functions of E2 required for the replication and maintenance of viral genomes. Furthermore, depletion of Brd4 using RNA interference has no effect on the association of BPV-1 and HPV-11 E2 proteins with mitotic chromosomes (Parish *et al.* 2006*a*). Put together, these data certainly suggest a role for Brd4 in the segregation of at least some papillomavirus types but its necessity in this process remains unresolved at present.

Other quests to isolate E2-associated cellular proteins have isolated new candidates that are involved in the segregation of papillomavirus genomes during mitosis. An interaction between HPV-16 E2 and the topoisomerase II-binding protein (TopBP1) has been described and shown to enhance the transcriptional and replication activities of E2 (Boner *et al.* 2002). More recently, TopBP1 has been hypothesized to be involved in viral genome segregation and shown to regulate the association of E2 with chromatin. E2 and TopBP1 colocalize during late mitosis and it is suggested that TopBP1 may be targeted by HPV-16 E2 to facilitate mitotic tethering (Donaldson *et al.* 2007).

Using a yeast two-hybrid system to screen a yeast cDNA library, an interaction between BPV-1 E2 and the DEAH family DNA helicase chromosome loss 1 (Chl1) was isolated (Parish *et al.* 2006*a*). Chl1 is required for the efficient segregation of chromosomes (Gerring *et al.* 1990) and studies on the human homologue, Chl-related 1 (ChlR1), have demonstrated a role in sister chromatid cohesion (Parish *et al.* 2006*b*; Inoue *et al.* 2007). The E2 proteins from BPV-1 and HPV types 11 and 16 have been shown to interact with ChlR1, demonstrating conservation of this interaction between genera. A mutant of BPV-1 E2 (W130R) has been isolated that no longer associates with ChlR1 but retains Brd4 binding, transcriptional activation and E2-dependent viral replication functions (Parish *et al.* 2006*a*). Although viral genomes that encode E2 W130R are able to transiently replicate in mouse fibroblasts, these genomes are not stably maintained, indicating that ChlR1 interaction is required for the maintenance of genomes over time and questions the necessity for Brd4 interaction in the tethering of PV genomes. This was confirmed by depletion of ChlR1, which disrupts the association of E2 with mitotic chromosomes. However, association is unaffected by the depletion of Brd4. Interestingly, E2 and ChlR1 do not colocalize during mitosis (Parish *et al.* 2006*a*), but a robust interaction is observed during the S-phase (A. Saade & J. L. Parish 2008, unpublished data) indicating that ChlR1 is required for the loading of E2 and viral genomes onto chromatin during the S-phase, possibly during cohesion establishment, and not for the direct tethering of genomes to chromosomes during mitosis. It remains to be seen whether ChlR1, Brd4 and/or TopBP1 act in concert to facilitate the segregation of viral genomes and whether these cellular proteins contribute to a cellular pathway required for the accurate segregation of chromosomes to daughter cells.

## 5. Conclusions

It is clear that many DNA tumour viruses have evolved complex mechanisms to ensure genome persistence (as summarized in table 1). There is much to be done to clearly define the exact mechanism each virus employs. Whether viruses that maintain genomes at low copy number have a different mechanism of genome tethering from those maintained at high copy number needs to be determined. Viruses that maintain few episomal genomes per cell may ensure that plasmids are not tethered to a single chromosome or multiple chromosomes that are segregated to one daughter cell. Future studies that elucidate such a mechanism will be fascinating. The importance of specific cellular proteins that are targeted to facilitate genome tethering must also be clearly defined. Nonetheless, the current depth of understanding allows us to see the common themes that have evolved to ensure the maintenance of episomally maintained genomes of vastly different sizes from diverse viral types. It is clear that all the viral types discussed in this review encode a protein that functions to tether viral genomes to host cell chromosomes during mitosis. This viral protein is a DNA-binding protein that specifically associates with consensus-binding sites within the viral genome, meanwhile associating with mitotic chromosomes. The association of these proteins with mitotic chromosomes can either be direct or indirect, mediated by association with host cell chromatin-bound proteins.

While the specific proteins each virus targets to mediate genome tethering are diverse, some common themes exist. For example, both KSHV LANA and papillomavirus E2 proteins have been shown to associate with bromodomain proteins, and both LANA and EBV EBNA1 proteins associate with components of the core histone. The involvement of cohesin in the segregation of viral genomes also seems to be consistent between different viral types. Cohesin associates with KSHV genomes, and EBV genomes are known to remain paired following replication. In addition, the tethering of papillomavirus genomes requires the cohesin establishment factor ChlR1, suggesting that this subset of viruses also target the cohesion establishment pathway.

Numerous studies have shown that the disruption of viral genome tethering during mitosis results in a loss of viral genomes over time. This opens the door for the design of novel antiviral therapeutics. In order for this to be successful, several factors should be carefully considered. Small molecules may need to be designed that affect only the tethering and not the replication or transcriptional functions of the viral protein(s) targeted. Such a small molecule will ensure that viral oncoproteins are held under strict transcriptional control and episomal genomes replicate normally. This will prevent any possibility of forced integration, as has been suggested for papillomaviruses (Lusky & Botchan 1985; Lambert & Howley 1988; Schiller *et al.* 1989; Taylor *et al.* 2003), although the potential risk of integration owing to aberrant replication needs to be further studied for a true link to be identified. Nonetheless, the design of novel and highly specific antiviral therapeutics that inhibit episomal genome tethering resulting in genome loss through rounds of cell division is an interesting concept and should be pursued.

## Figures and Tables

**Table 1:** Summary of the DNA viruses that actively segregate genomes during mitosis and the reported mechanisms by which this is achieved.

virus	genome size (base pairs)	viral protein required for genome tethering	chromosomal association pattern during mitosis	suggested associated cellular protein(s) that facilitate segregation
EBV	165 000	EBNA-1	paired foci randomly associated with chromosomes	Histone H1 and EBP2
KSHV	140 000	LANA	random speckles	MeCP2, DEK, Brd2/4 histones H1, H2A and H2B, NuMA
papillomavirus	8 000	E2	random speckles or foci near spindle attachment region	Brd4, ChlR1 and TopBP1
